# CCMRI: a classification and curated database of climate change-related microbiome studies

**DOI:** 10.1038/s41598-026-51914-z

**Published:** 2026-05-06

**Authors:** Alexios Loukas, Konstantinos Kalaentzis, Nefeli Kleopatra Venetsianou, Christina Damianou, Savvas Paragkamian, Vincenzo Lagani, Lars Juhl Jensen, Evangelos Pafilis

**Affiliations:** 1 Institute of Marine Biology, Biotechnology and Aquaculture, Hellenic Centre for Marine Research, P.O.Box 2214, 71003 Heraklion, Crete, Greece; 2 Hydrobiological Station of Rhodes, Hellenic Centre for Marine Research, Rhodes, Greece; 3 Institute of Marine Biological Resources and Inland Waters, Hellenic Centre for Marine Research, Anavyssos Attikis, Greece; 4 Department of Biology, University of Crete, Heraklion, Crete, Greece; 5 Biomedical Sciences Division, King Abdullah University of Science and Technology, Thuwal, Saudi Arabia; 6 Institute of Chemical Biology, Ilia State University, 0162 Tbilisi, Georgia; 7 Novo Nordisk Foundation Center for Protein Research, University of Copenhagen, Copenhagen, Denmark; 8 ZS Discovery, Kongens Lyngby, Denmark

**Keywords:** Climate change, Semi-automated curation, Microbiome study classification, Large language models, Metagenomics, Manually curated corpus, Computational biology and bioinformatics, Ecology, Ecology, Environmental sciences, Microbiology

## Abstract

Climate Change (CC) is reshaping all ecosystem processes and structures. Microbial data provide valuable insights into how microbial processes contribute to CC and how CC, in turn, alters microbial communities. However, the growing volume of environmental genomics data makes identifying CC-related records challenging. The Climate Change Metagenomic Record Index (CCMRI) has been developed to harvest metagenomic/microbiome records pertaining to CC and to provide researchers with a curated database of CC-related microbiome studies (https://ccmri.hcmr.gr). To guide interpretation, the database’s 169 metagenomic studies have been labelled according to their relation to CC as CC-caused, CC-causing, and CC-mitigating. They have also been annotated with the CC phenomena they explore, like methane production, temperature rise, permafrost thawing, greenhouse gas emission, methanotrophy, and ocean acidification. To ease navigation, they have also been classified according to their biome as aquatic, terrestrial, host-associated, and engineered. The CCMRI database was initially constructed through manual curation of all aquatic and terrestrial studies in the MGnify resource. It was then expanded with the help of the CCMRI curation-assistant system. This leveraged Large Language Models to scan the remaining MGnify studies, filtered them for relevance, and proposed candidates for inclusion. With a recall greater than 90%, the system achieved high accuracy in identifying CC-related studies. The final decisions on CC-relatedness and categorization were performed by a human curator. This approach combines the efficiency of automation with human oversight and greatly reduces the curation effort, ensuring sustainability and scalability.

**Supplementary Information:**

The online version contains supplementary material available at 10.1038/s41598-026-51914-z.

## Background

Climate change is reshaping life on Earth. Traditionally, climate science has focused on abiotic components, such as temperature, greenhouse gas concentrations, atmospheric humidity, ice cover, and sea level^1^. These changes are driven primarily by anthropogenic activities, including fossil fuel burning, deforestation, and agricultural production^2^, which in turn lead to global warming, aridification, biodiversity loss, eutrophication, and other environmental impacts. However, the biotic components are equally fundamental^3^.

Microorganisms, which dominate biomass, pervade ecosystems and drive the nutrient cycle^4^, both affect and are affected by climate change (CC)^5^. Historically, microbial activity has shaped the atmosphere, including the oxygenation driven by cyanobacteria billions of years ago^6^. Today, climatic changes can disrupt microbial processes, potentially amplifying environmental changes through feedback loops and provoking irreversible shifts^3,6–8^. Growing efforts aim to harness microbial processes addressing CC, involving mitigation strategies, with interventions such as biofertilizer usage or microbial inoculants combined with biochar, shown to reduce greenhouse gas emissions and enhance carbon sequestration^9^. Advancing these strategies requires effective discovery and analysis of climate-related metagenomic/microbiome studies to identify and develop microbial solutions.

Environmental metagenomic data provide a new conceptual framework for studying microbial contributions to climate dynamics^3^. Global resources, such as MGnify^10^, MG-RAST^11^, and JGI/IMG^12^, provide access to indexed metagenomic datasets from various experiments, including amplicon and shotgun metagenomics. These platforms allow text- or sequence-based searches and browsing by predefined hierarchies, including that of environment types (biomes).

**Fig. 1:**
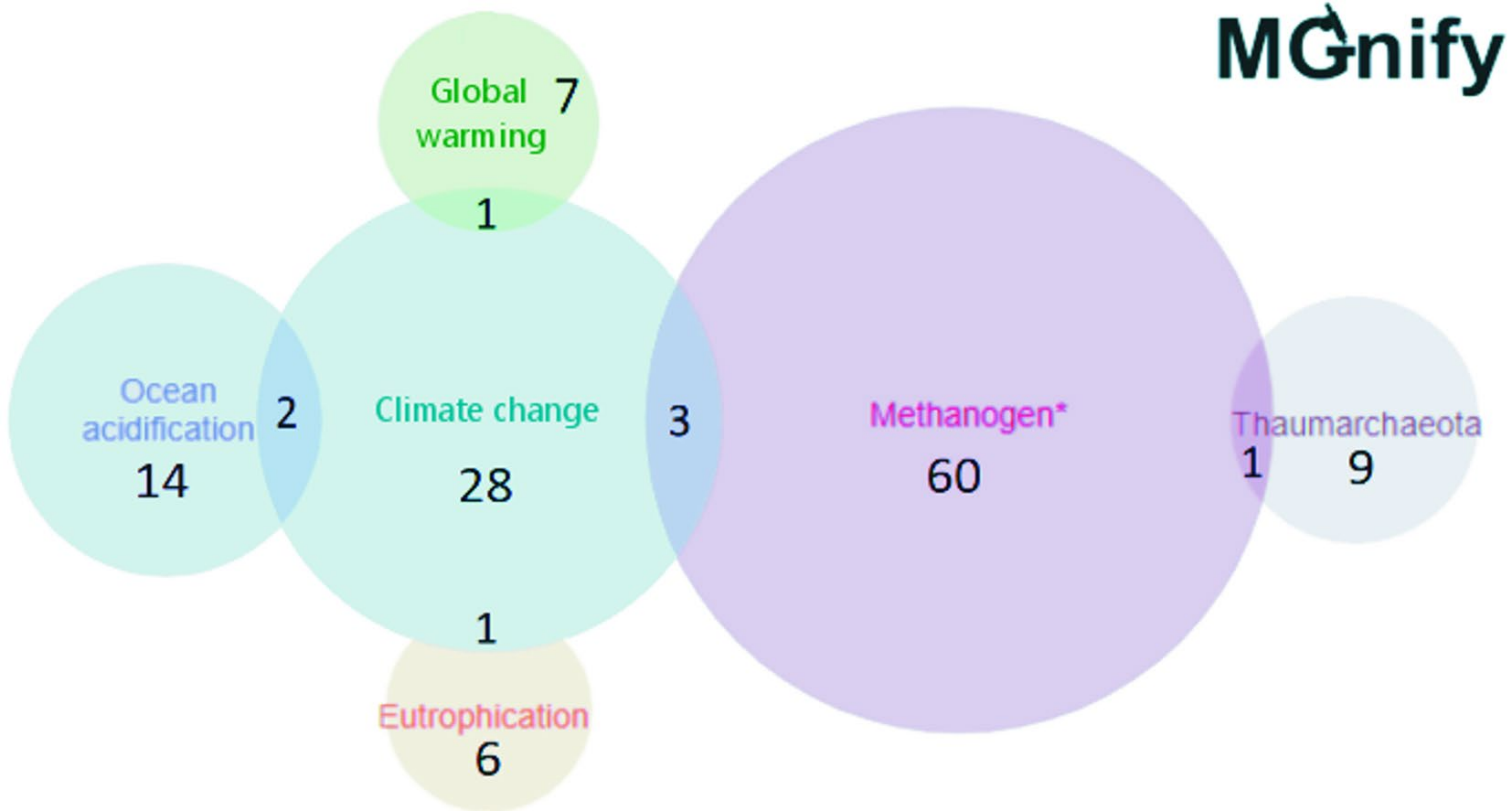
Number of metagenomic/microbiome studies retrieved by CC-related queries in the EBI MGnify resource. Simple queries such as “climate change” or “global warming” capture only part of the available information (queries as of 24 July 2025). For example, searching for “climate change” returns 35 studies. Other related terms, including “global warming”, “ocean acidification”, and “eutrophication”, mostly retrieve different sets of studies with very little overlap. Additional queries such as “Methanogen*” (60 studies) and “Thaumarchaeota” (9 studies) indicate that an even larger pool of relevant studies can be identified using domain or taxon-specific keywords that are not explicitly linked to CC terminology. Overall, this highlights that many relevant studies are indexed under only a limited number of specific keywords, making the comprehensive discovery of CC-related studies challenging.

Despite these resources, locating datasets specifically relevant to CC remains a tedious and fragmented process. As shown in Fig. 1, many relevant datasets are listed under only one term, which makes it difficult to retrieve all available data with a single query and highlights the challenge of comprehensive discovery. This difficulty is compounded by the way metadata are reported in metagenomic/microbiome datasets^13^. As essential information, metadata are often incomplete, inconsistent, or hidden within publication text, limiting automated discovery and reuse. Mitigation techniques such as enriching records with information from other databases or by combining information from multiple resources can help fill the gaps and improve discoverability.

Tools for enriching metagenomics metadata from the literature^14^ are available. Effective methods for identifying, curating, and sharing climate-change-related studies are still needed to fully exploit metagenomic resources to this end.

Here, we present the Climate Change Metagenomic Record Index (CCMRI), a semi-automated classification and curation system that successfully identifies CC-related metagenomic/microbiome studies and accelerates the curation process. To develop and evaluate the system, we constructed a corpus from textual metadata extracted from MGnify, covering both terrestrial and aquatic studies, and split it into training and evaluation sets. The training set was used to explore classification methods utilizing both machine learning (ML) methods and large language models (LLMs), while the evaluation set assessed their performance and determined the optimal solution. Our results show that, in our case, LLMs outperformed standard ML approaches, effectively triaging studies and saving significant time for curators. This system was integrated into a web-based repository^15^, where all identified CC-related studies are displayed and organized according to relevant categories. Finally, an internal curation platform optimizes the curation process.

## Related work

Early methods of scientific text classification systems relied on rule-based approaches, using predefined rules and heuristics to classify and extract metadata from structured documents^16,17^. While straightforward to implement, these early systems require a lot of preliminary setup, often lack flexibility, and cannot address the variability of natural language in scientific publications^18^.

With the advent of ML, systems began to learn patterns from annotated datasets, improving their ability to generalize across different document types^19^. Techniques such as logistic regression and XGBoost were applied to classify and extract metadata from scientific literature. These ML-based approaches demonstrated improved performance over traditional rule-based systems, especially in handling the complexity and variability of scientific texts^19–21^.

Recent advancements have demonstrated the use of LLMs to extract metadata from scientific texts, as explored in prior work employing GPT-3 and Llama 2^22^, but also BERT^23,24^.

These models, trained on a vast text corpus, can understand and generate human-like text, allowing for more nuanced extraction of metadata attributes. Studies have shown that LLMs can effectively extract biological terms and metadata from scientific publications, enhancing the usability of databases like BioSample^25^.

LLMs are also being applied to microbiome research to analyze complex genomic and protein sequence data, including tasks such as predicting biosynthetic gene clusters and modeling metagenomic datasets^26^. They have also been used to support FAIR-compliant microbiome databases, making data more accessible and user-friendly and demonstrating their potential for automated data curation^27,28^. Finally, LLMs have been used to classify microbiome samples/sequences into broad ecological environments^29^.

Considering the advances described above, LLMs, such as Llama 3 and GPT-4, are worthwhile for document classification. In particular, they were shown to outperform traditional ML methods under certain conditions^30–32^. Exploring both standard ML methods and LLMs could help establish a classification system. Each approach may offer effective solutions to identify CC-related studies within a large corpus of microbiome datasets.

## Results & discussion

### Workflow

**Fig. 2:**
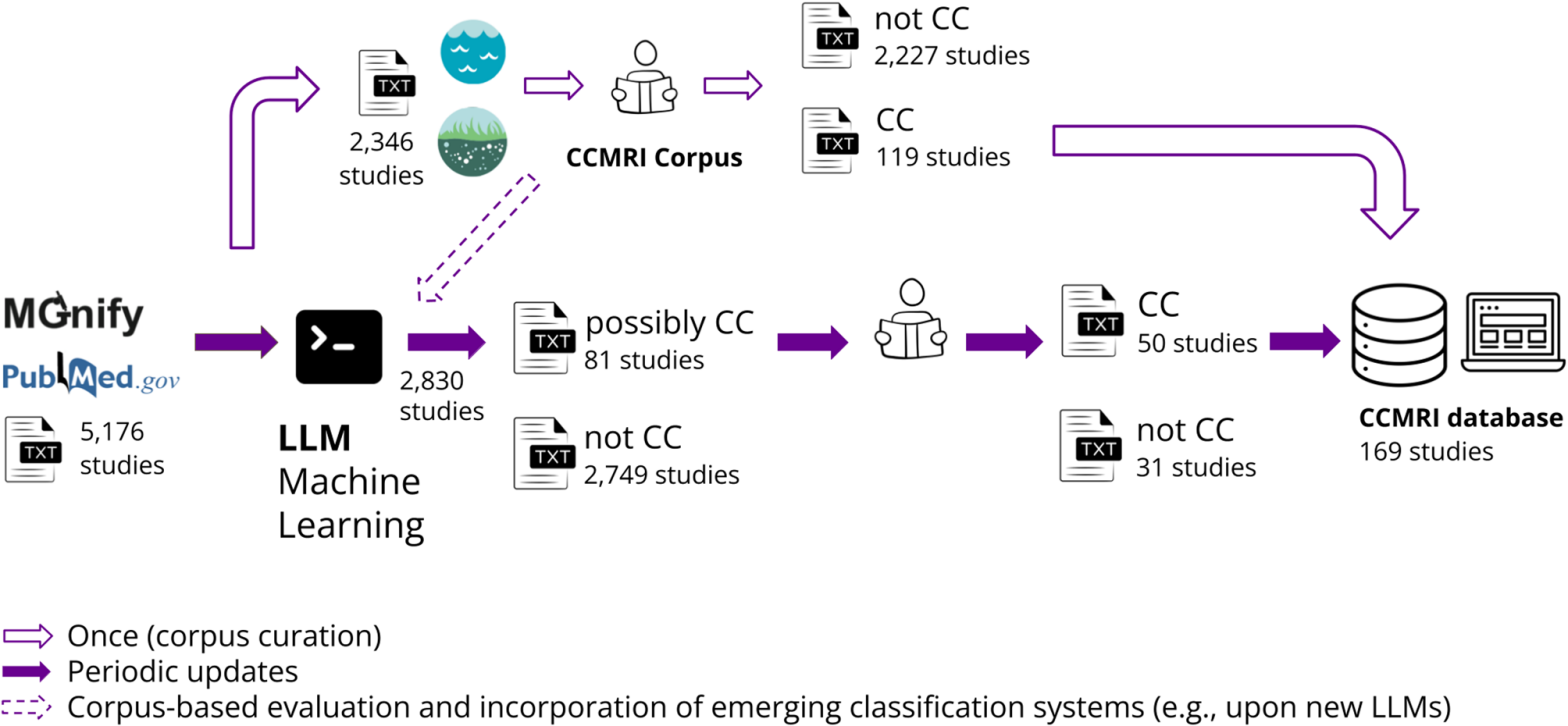
The diagram illustrates the development and application of the CCMRI workflow. The upper path shows the creation of the CCMRI corpus, integrating textual data from MGnify with PubMed abstracts, resulting in a curated set of 2346 studies from aquatic and terrestrial environments. Of these, 119 were identified as CC-related through curation, which required six person-months. This corpus served as the basis for training and validating the machine learning (ML) and large language model (LLM) classifiers. The lower path demonstrates the LLM classifier applied in a semi-automated fashion to the 2830 remaining MGnify studies. The classifier flagged 81 potential CC-related studies for human review. Following one week of manual curation, 50 out of 81 studies were validated as CC-related, representing a 30-fold acceleration in curation efficiency compared to the manual curation of the corpus. Confirmed studies were also incorporated into the CCMRI database and made available through the web portal. (Icon attribution: Noun Project, see Supplementary Material).

To support the CCMRI project, we developed a structured workflow (Fig. 2) that integrated both automated and manual procedures. It encompassed the retrieval of metagenomic study texts from MGnify, the creation and curation of the CCMRI corpus, the development of ML and LLM classification modules to identify potentially CC-related studies from larger datasets, and the subsequent manual review of candidate studies via the curation platform. Curated and finalized studies were then organized in the CCMRI portal, enabling structured access to climate change-related information. The following sections detail the outcomes generated through the application of this workflow, including corpus construction, classifier performance, semi-automated curation, and database population.

### CCMRI corpus

The CCMRI corpus formed the basis for populating the database with the first set of CC-related studies and played a central role in evaluating classification performance. The training portion of the corpus enabled the development of consistent curation guidelines and served as a reference benchmark for comparing ML models and LLMs. Approximately 5.1% of the corpus studies (119 in total) were identified as CC-related, with 75 in the training set and 44 in the evaluation set.

The CCMRI corpus was annotated by two curators, adhering to the guidelines specified in the Materials and Methods section. This process took a total of six person-months. To assess the Inter Annotator Agreement (IAA), 95 studies were reviewed independently by both curators. Out of the 95 studies, 93 received identical labels, resulting in an IAA (Cohen’s kappa score) of 82%, indicative of almost perfect agreement^33^ (Supplementary Table S1).

### Performance comparison of study classification methods

Study classifiers form the foundation of the CCMRI pipeline (Fig. 2). To identify the most suitable system for our application, we compared a logistic regression model with the top five LLMs in terms of recall, focusing on which method could best serve as the study classification system.

**Fig. 3:**
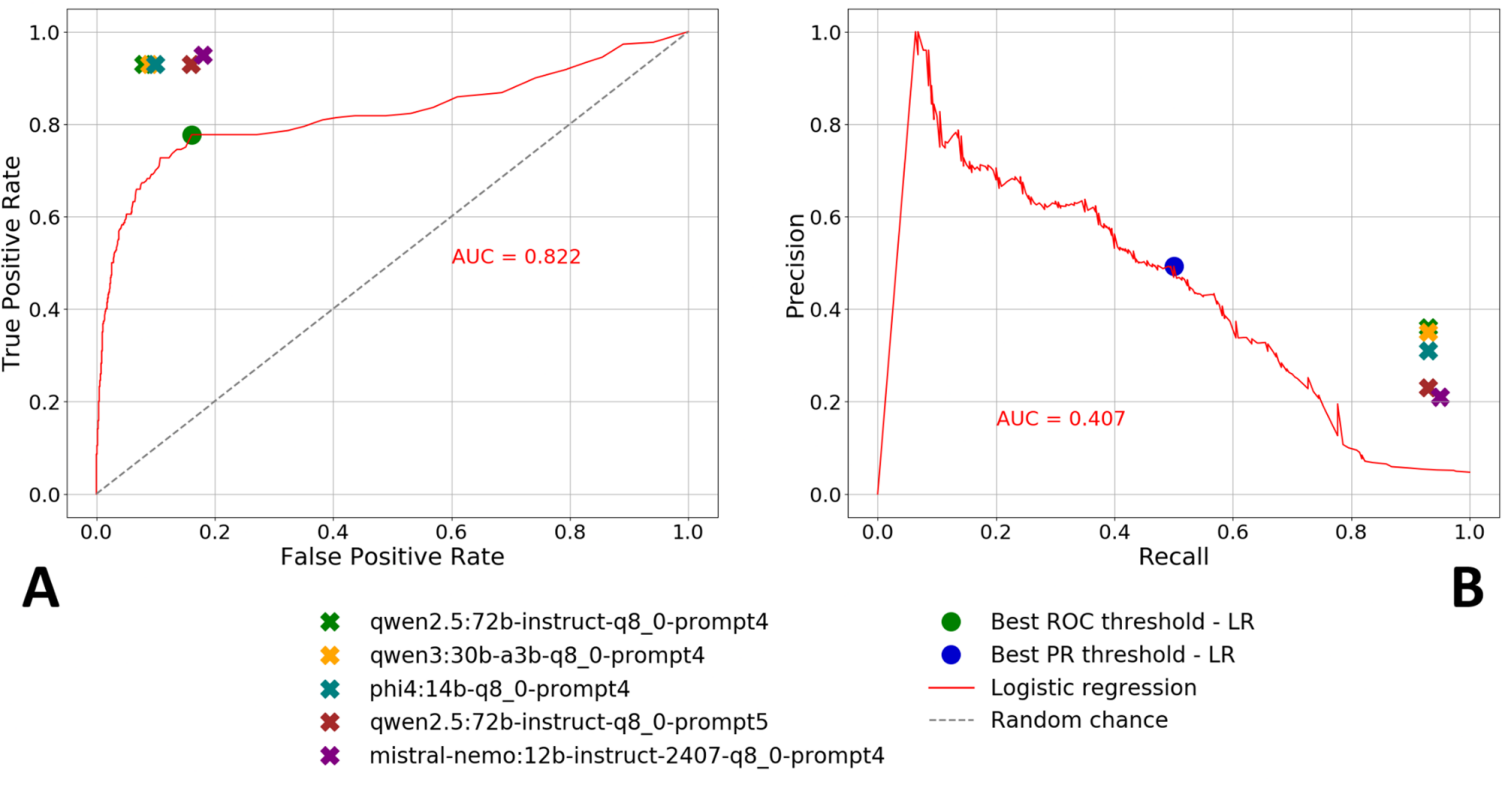
Receiver Operating Characteristic (ROC; **Α**) and Precision-Recall (PR; **Β**) curves for the combined held-out dataset, comparing the performance of all classification systems. The logistic regression model (red curve) was evaluated through threshold analysis, computing scores across multiple decision thresholds (up to n-1, where n is the number of MGnify studies in the dataset). The optimal ROC and PR thresholds for logistic regression (LR) are marked with green and fluorescent green circles, respectively. The LLM models are represented as individual points, each corresponding to their performance metrics (aggregated over three runs per study using majority voting). These results provide a comparative overview of the classification capabilities of both traditional ML and instruction-tuned LLM classifiers.

As shown in Fig. 3, the best-performing ML model (logistic regression) trained on the combined super-vector dataset is plotted alongside the top five LLM-based systems in terms of recall. The LLM predictions are represented as single points, while the logistic regression curve results from a threshold sweep across all classified MGnify studies using *n-1* thresholds, where *n* is the number of studies, meaning that 938 studies in the combined dataset yield 937 thresholds. Each LLM point represents the result of a majority voting procedure over five independent runs of the model (three out of five), ensuring more stable and consistent classification outputs.

In the Precision-Recall (PR) curve (Fig. 3B**)**, all LLM models consistently exhibit high recall (93–95%) but relatively low precision (21–36%), indicating their tendency to classify more studies as CC-related. This behavior is particularly valuable in our context, where recall is prioritized over precision; our goal is to avoid missing potentially relevant CC-related studies, even at the cost of having to manually inspect more false positives (Fig. 2, purple-filled-arrow path).

Conversely, the ML model demonstrates a typical trade-off: at lower decision thresholds, recall is high but precision is low, and as thresholds increase, precision improves while recall declines. This gradual transition is reflected in the PR curve’s shape (Fig. 3).

In the ROC curve (Fig. 3A), which considers both sensitivity and specificity (Supplementary material: B. Evaluation metric definition), all tested LLMs outperform the logistic regression baseline across most of the curve, indicating superior overall classification capability. Similar performance trends were observed in both the terrestrial and aquatic subsets (Supplementary Figures S1 and S2), where LLMs consistently achieved higher true positive rates than ML at comparable or lower false positive rates. Moreover, by comparing the two subset results, LLMs demonstrate better performance in the aquatic studies. This may be linked to the composition of the subset and agrees with the curators’ remark that—due to clear-cut cases—aquatic studies were easier to curate than the terrestrial ones.

These results underscore the practical value of LLMs for our task, especially in high-recall scenarios. While precision remains modest, the ability of LLMs to generalize without explicit training and still match or exceed traditional methods reinforces their potential for integration into semi-automated or curator-assisted classification pipelines. In contrast to traditional methods, which often rely on extensive manual feature extraction and domain-specific engineering, LLMs significantly reduce human effort. Their scalability and adaptability make them applicable across diverse datasets and evolving domains, providing flexibility that earlier methods cannot achieve as effectively. A notable limitation, however, is the computational demand: larger LLMs require GPUs for efficient processing, and CPU-only deployments can become prohibitively slow for high-volume or real-time applications.

The majority-voting strategy (three out of five) helped to improve the reliability of the evaluation metrics. It has been shown that this approach improves stability and reduces variance in classification outputs^34,35^.

Among the evaluated LLMs, while Qwen2.5 72B demonstrated slightly superior precision and ROC performance, the Qwen3 30B A3B model was ultimately selected for integration into the classification pipeline. This decision was driven by practical considerations: Qwen3 30B A3B offered competitive results while being significantly more computationally efficient than the larger Qwen2.5 72B parameter model. Its balance of performance and resource requirements makes it a more scalable and sustainable choice for large-scale screening and future deployment.

It is important to note that the choice of prompts plays a crucial role in LLM performance. In our experiments, using the same model with different prompts (Supplementary material: D. LLM full prompts) produced different outcomes. This can also be observed in Fig. 3, where in the ROC curve, the Qwen2.5 72B model yields a higher false positive rate when using prompt5. Additionally, most models in Fig. 3**,** are run with the same prompt and yield high recall while ranging from 12–72 billion parameters, which enhances the role of the prompt. These results highlight how careful prompt design can substantially influence model effectiveness. These findings are in line with Gaio et al*.*^29^, who showed that more explicit, task-specific prompts improved performance, whereas changes in model architecture or size had a smaller effect.

### CCMRI database

The CCMRI database currently contains 169 studies: 119 manually curated from the CCMRI Corpus, and an additional 50 identified through the LLM-based classification of the 2,830 MGnify studies. All studies in the database are categorized using multiple dimensions: their CC relevance (i.e., CC-caused, CC-causing, or CC-mitigating), associated biome (e.g., marine, soil, freshwater), and observed environmental or ecological phenomena.

**Fig. 4:**
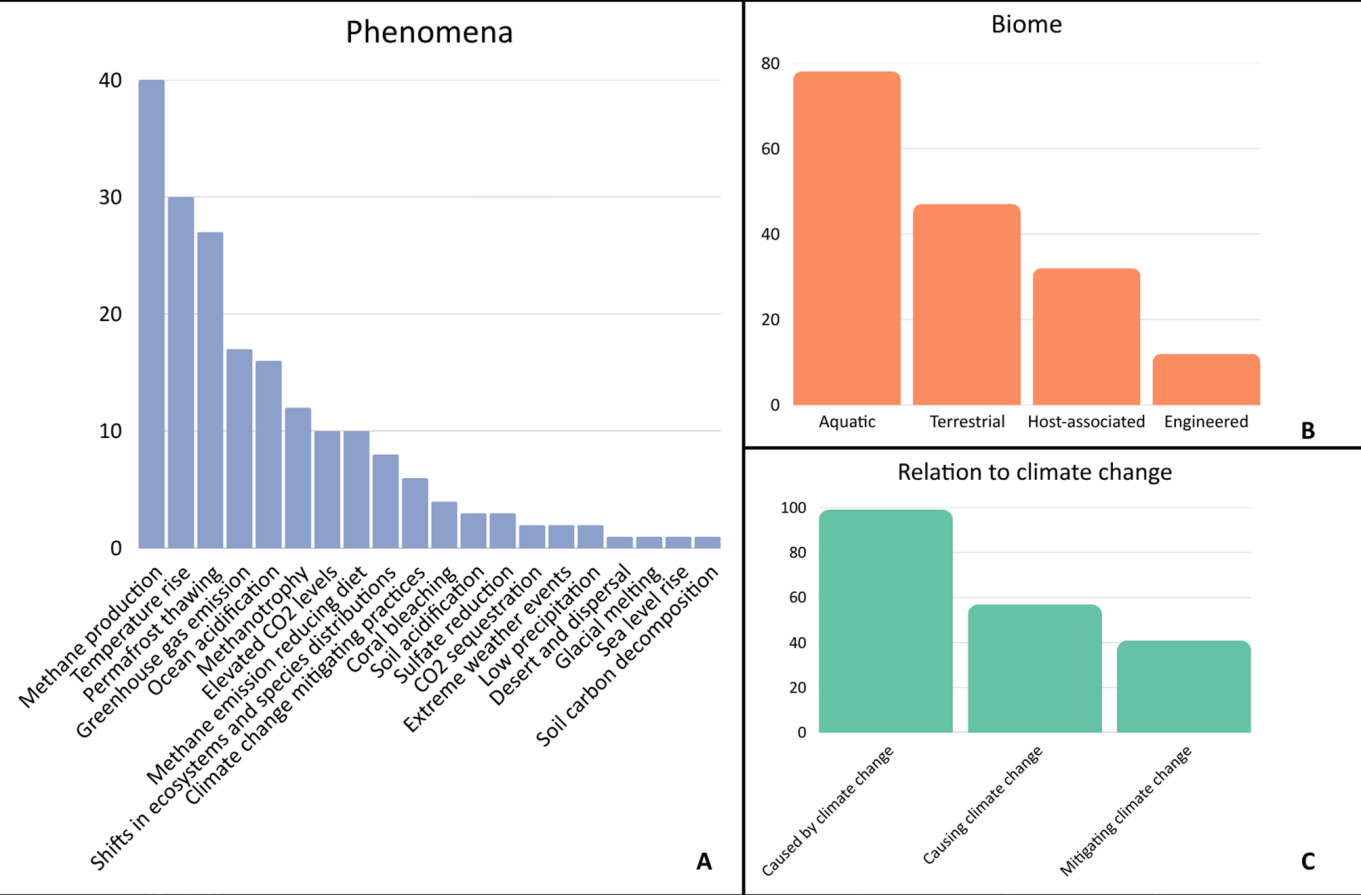
Distribution of CC-related studies in the CCMRI database according to categorization tags. (**A**) Phenomena reported in studies, with methane production accounting for 40 studies, temperature rise for 30 studies, and permafrost thawing for 27 studies as the most frequent tags, followed by greenhouse gas emissions, ocean acidification, and other microbial processes at lower frequencies. (**B**): Biomes represented in studies, showing a dominance of aquatic systems (78 studies, including 61 marine and 12 freshwater ones), with fewer terrestrial (47), host-associated (32), and engineered (12) studies. (**C**) Relation of studies to climate change, with the majority causally linking microbiomes and climate change as caused by CC (99 studies), while fewer report microbes as causing climate change (57 studies) or mitigating it (41 studies).

In terms of CC relevance, most studies are classified as caused by climate change (Fig. 4C**)**. Aquatic biomes contribute the largest portion of studies, followed by terrestrial biomes (Fig. 4B). The most frequently reported phenomena include methane production, temperature rise, and permafrost thawing (Fig. 4A). The above-mentioned categories have also been integrated in the CCMRI web portal to support search and filtering functionality, allowing users to easily explore the database content.

**Fig. 5:**
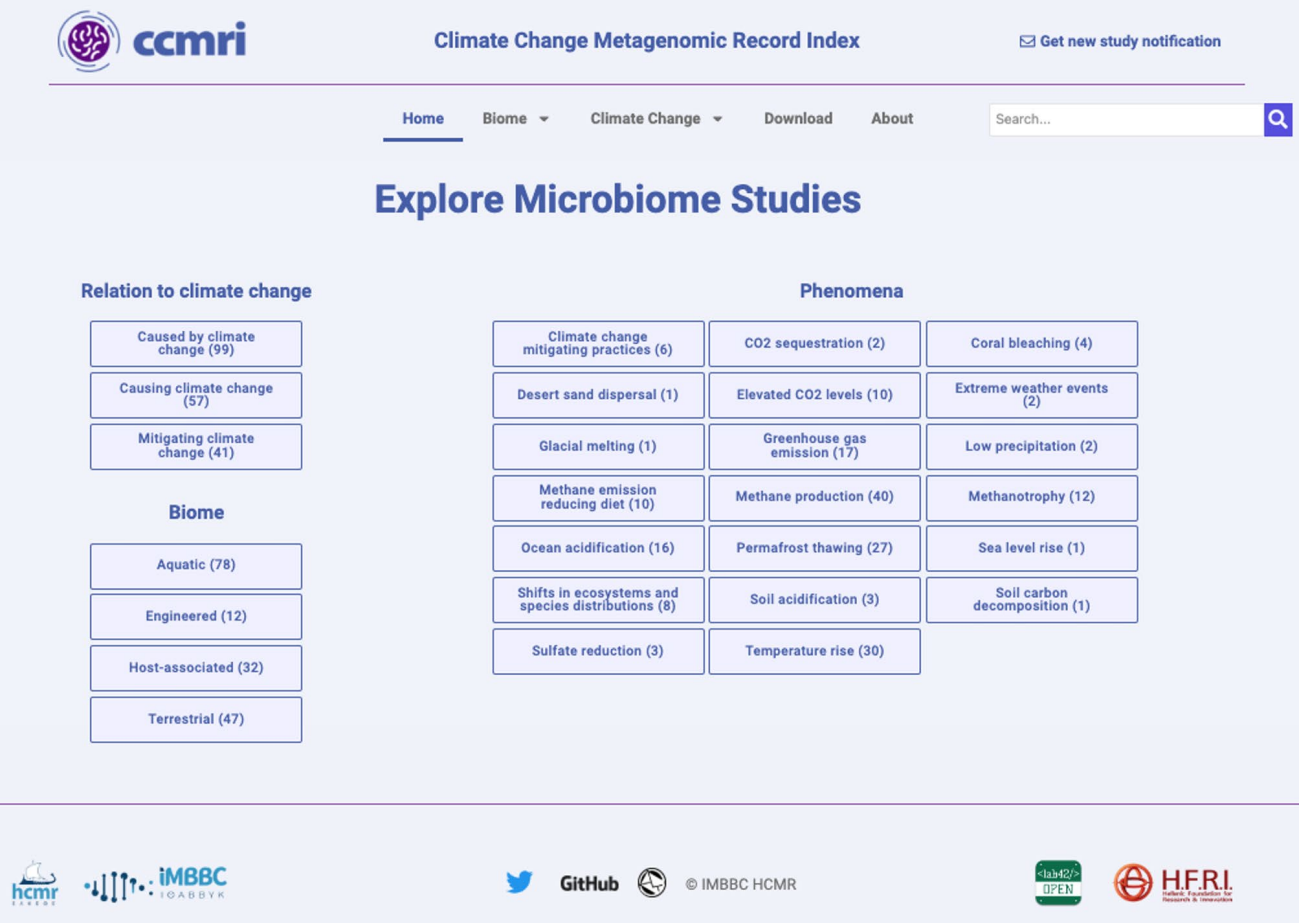
The home page of CCMRI. The portal provides navigation to studies, browsing by CC relation, biome, and environmental phenomena, with study counts displayed for each category.

**Fig. 6:**
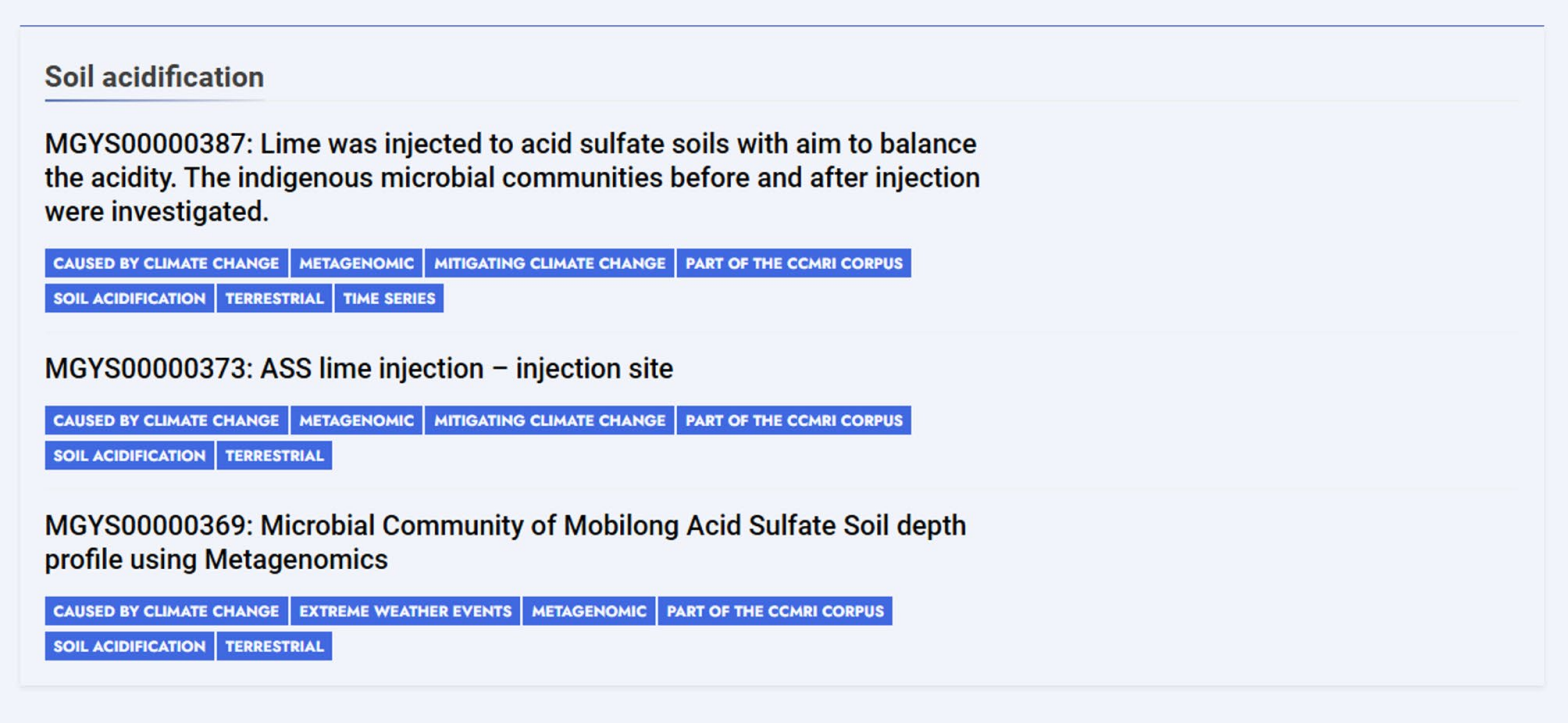
List of studies associated with the Soil Acidification phenomenon. The CCMRI portal displays studies grouped by related environmental phenomena, with each entry showing the MGnify record ID, study title, and associated categories. The latter are clickable to support navigation further.

**Fig. 7:**
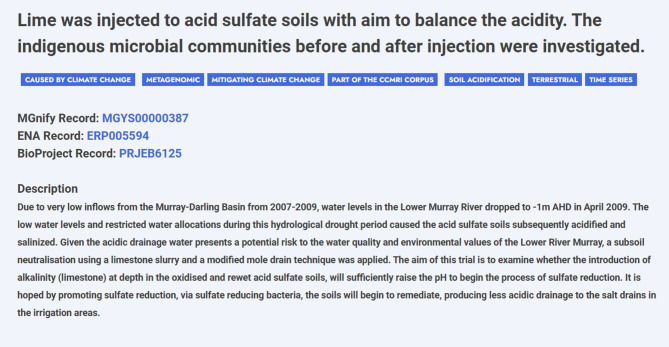
Single study record from the CCMRI web portal. Each entry includes the MGnify record ID, description, biome, CC annotations, and study analysis type (e.g., amplicon, metagenomic). Links to associated NCBI BioProject and ENA records are also provided. Studies involving samples collected over time are categorized as “time series”. When available, publication details such as PubMed title, ID, and abstract text are additionally displayed (not shown in this example).

Building on this structure, the CCMRI web portal^15^ provides a publicly accessible interface to the CCMRI database. The homepage (Fig. 5) displays the number of studies available within each category, providing an at-a-glance overview of the current database content. The platform supports hierarchical navigation (menu bar), text-based search, and category-based browsing by clicking in any of the boxes. Figure 6, for example, shows microbiome studies related to soil acidification. For each study, categorization tags are highlighted, along with MGnify ID, description, biome, CC annotations, study analysis type (e.g., amplicon, metagenomic), links to associated NCBI BioProject, ENA records, as well as related PubMed publications (if any) (Fig. 7).

To ensure the resource remains up to date, the CCMRI database is periodically expanded with newly curated studies. Users can subscribe to an email notification service, without creating an account, to receive alerts when new studies become available. An internal curation platform supports the annotation of new studies, which is presented in the next section.

### Database curation and platform update

As the volume of public metagenomic data grows^10^, efficient curation becomes increasingly important. Keeping the CCMRI database current relies on a streamlined curation process.

The cooperation of the CCMRI classification LLM system with the web-based curation platform greatly accelerates the identification and categorization of relevant studies, enabling regular database updates that would otherwise be prohibitively slow with manual curation alone.

Within four hours, the LLM classifier analyzed the remaining 2830 MGnify studies (i.e., except the CCMRI corpus ones) and flagged 81 as potentially CC-related (Methods, LLM-based classification of MGnify studies). After a person-week of manual review, 50 of these were confirmed and published in the CCMRI database/platform. In comparison, the manual curation of the original corpus (2346 studies) required approximately 25 person-weeks.

Whereas 5.1% (119 out of 2346) of the manually curated studies are CC-related, the same is true for only 1.8% (50 out of 2830) of the studies subject to semi-automated curation. The reason for this difference is that 2830 MGnify studies represent a variety of environments beyond those covered in the aquatic and terrestrial categories, e.g., host-associated microbiomes. A breakdown of the 50 aforementioned studies shows that 32 of the 50 are host-associated studies (excluding human as the host organism), and 12 are from engineered environments. The last six studies are five aquatic and one terrestrial, retrieved after the corpus freeze.

In terms of curation effort, annotating all 2830 studies manually would have required roughly 30 person weeks, assuming a manual curation speed of ~ 94 studies per person week (i.e., 2346 studies curated over 25 person weeks). Thus, by pre-filtering with the LLM classifier, the effective annotation workload was reduced from 30 person weeks to just one, representing a 30-fold acceleration in curation efficiency. The fact that the employed LLM (Qwen3 30B A3B) achieved a recall of 93% (Fig. 2, purple-filled-arrow path) strengthens the credibility of the CCMRI approach and demonstrates its practical feasibility. Comparable levels of recall have also been reported in LLM-based related biomedical research^36^.

While the CCMRI system’s precision of 35% is obviously not good enough to eliminate the need for manual validation, high recall is what matters most in a curation support system. Similarly, E. Niyonkuru et al*.*,^37^ have also prioritized recall over precision in their LLM-based curation system. It is inherently hard to get good precision for the task at hand, since the studies of interest are rare (only 5.1% of the studies in the CCMRI corpus are CC-related).

One important question is: to what extent does the LLM justification help the curators? Only one person curated the 81 studies candidates to be CC-related, therefore IAA/quantitative assessment was not possible. Qualitative assessment showed that LLM’s justifications were useful in some instances, stating the obvious otherwise. As the LLMs improve, their justification usefulness is expected to increase as well. This is one more reason for an easy-to-update approach in the CCMRI architecture (Fig. 2 (dashed arrow), see also below).

The curation platform provides curators with the LLM’s predictions and justification text, allowing them to review the reasoning behind each classification, flag potential errors, and gather insights for future improvements. Curators access the system through WordPress accounts with Moderator privileges. Each study entry displays its metadata (ID, title, biome, description, URL), along with the LLM-generated justification explaining why it was flagged as CC-related. A comment field enables curators to document decisions, while standardized curation guidelines ensure consistency in evaluation (Methods, CCMRI corpus). When a study is verified as CC-related, it is annotated using a structured set of checkboxes covering biome type, CC relation type (CC-caused, CC-causing, or CC-mitigating), and associated environmental phenomena such as coral bleaching, sea level rise, or permafrost thawing. These annotations are implemented using hierarchical post categories that capture both the biome, CC relation, and related phenomena. They are managed by the curators (e.g., categories can be edited, added, or removed). Upon a change, the CCMRI portal content is updated automatically. Examples of the manual inspection/curation platform interface are shown in Supplementary Figure S3 and Supplementary Figure S4.

After each periodic update, new possible CC-related study suggestions and a manual inspection/curation cycle (Fig. 2), validated studies are incorporated into the CCMRI database and published on the web portal, ensuring that the platform reflects the most up-to-date collection of climate change-related microbiome research. In addition, the modular CCMRI implementation (Fig. 2) allows for novel classification modules to be employed as they emerge. When a new LLM model becomes available, it is possible to evaluate it based on the CCMRI corpus, and in the case of a performance gain, to replace the previous ones. Such a modular, semi-automated classification streamlines study processing and establishes an infrastructure that curators can rely on for sustainable, large-scale updates.

Additionally, the long-term sustainability of CCMRI will benefit from improvements in study-level metadata, such as more informative titles and descriptions, and more consistent links to PubMed records. Repositories such as the National Microbiome Data Collaborative (NMDC)^38^ and Metalog^39^ offer higher-quality metadata that can support these improvements. While CCMRI currently focuses on a subset of metadata types, future extensions could leverage additional metadata dimensions as they become more standardized and widely available.

## Conclusions

The CCMRI web portal provides microbiome researchers focusing on CC and microbial processes with a centralized resource to access and explore all relevant studies in one place. As the number of CC-related microbial studies continues to increase, the database will expand accordingly, offering broad coverage and deeper insights. An important advantage of the system is the rapid curation enabled by LLM-based classification, as the time required for curation is dramatically reduced compared to fully manual approaches, allowing accelerated integration of new studies. Improvements in metadata standards will further enhance the richness and quality of available information, supporting more comprehensive analyses.

## Methods

### Data retrieval

To facilitate the analysis, data from MGnify is downloaded and organized. For each study, a set of metadata fields is saved in text files, including key details such as study ID, study name, study description, BioProject ID, secondary accession, last update date, and biome information. To enrich the dataset, pertinent PubMed abstracts (along with PubMed ID, title, and publication year) are downloaded from the PubMed FTP server and incorporated into our textual data files. PubMed abstracts associated with 16 or more MGnify studies were considered non-specific and excluded from the analysis. These abstracts typically describe resources, databases, or computational platforms rather than study-specific findings. This filtering ensures the textual data used for classification is relevant and informative.

To improve efficiency, the system is designed to recognize previously downloaded studies, avoiding redundant data collection by retrieving only new studies and their corresponding records/information. All downloaded content is organized into folders named after each study’s unique MGnify ID for easy access and management. These IDs are also included in the filenames containing the obtained text.

### CCMRI corpus

The CCMRI corpus, a dataset of 1,761 aquatic and 585 terrestrial microbiome studies from MGnify (2,346 studies in total), was curated by two annotators. To this end, detailed curation guidelines were developed and made available (**see Data Availability**) to ensure consistent labeling and reproducibility across the corpus.

The manual curation was performed by inspecting the study titles and descriptions in MGnify. The titles and abstracts of any linked publications in PubMed were also examined.

Studies were annotated as CC-related if they addressed either the influence of CC on microbial communities (CC-driven impacts on microbiomes; CC-caused) and/or the contribution of microbial communities to CC (microbiome-mediated processes contributing to CC; CC-causing) and/or microbiome-related mitigation strategies against CC (CC-mitigating). The phenomena relating to a study were recorded too. Examples of CC-causing phenomena included greenhouse gas emissions and methane production, while among the CC-caused phenomena were temperature rise, ocean acidification, extreme weather events, and permafrost thawing. CC-mitigating strategies involved methanotrophy and sulfate reduction.

To check how consistently the two curators applied the annotation criteria, they independently reviewed the same set of 95 studies. We then measured how closely their decisions agreed, that is, whether each study was considered CC-related or not, using Cohen’s kappa metric (see CCMRI Corpus in the Results & discussion section, Supplementary Table S1).

### Dictionary construction for NER

Identifying terms relevant to climate change is a critical first step for Named Entity Recognition (NER) and subsequent ML training for classification. To find the mentions of these terms in the obtained text, we used the JensenLab Tagger^40,41^, a dictionary-based NER tool, which has previously been applied for the detection, among others, of taxonomic names^42^ and environmental descriptors^43^.

To support NER in this study, five sub-dictionaries relevant to CC were constructed (Table 1), including those covering generic CC-related terms, CC-related environmental processes, and CC-related taxa (e.g., methanogens). Additional sub-dictionaries included negative words, which are terms frequently used in other contexts or expected less in microbiome studies, such as “environmental” or “human”, and time-frame words, like “decade,” that indicate that the study involves comparisons across time intervals, like sampling the same location every few years to observe changes over time.

**Table 1:** The structure of the CCMRI dictionary (along with the term sources) used for NER. All five sub-dictionaries are shown.

Entity name	Entity code	Number of entities	Number of names	Ontologies/Hierarchies used
Process	− 83	550	1978	ENVO, GO, BFO
Generic keywords	− 84	1579	4729	CSO
CC-related taxa	− 2	95,033	95,033	NCBI Taxonomy ID
Negative words	− 85	2	2	–
Time-frame words	− 86	12	29	–

To standardize terminology and improve interoperability, the dictionaries were aligned with relevant ontologies whenever possible. For example, environmental processes and generic CC terms were linked to various ontologies, i.e. the Environment Ontology (ENVO)^44^ and Gene Ontology (GO)^45^, the Basic Formal Ontology (BFO)^46^, and the Climate System Ontology (CSO)^47^. However, for some categories, including CC-related taxa and certain generic or negative terms, formal ontology mappings were not always available. For such instances, mappings to the corresponding NCBI Taxonomy^48^ records, and manually curated term lists were used respectively (Table 1).

This approach ensured broad coverage while maintaining as much semantic structure as possible. Finally, for implementation in the pipeline, the five sub-dictionaries were merged into a single integrated file, ensuring that all relevant terms could be accessed within a unified CCMRI dictionary.

### Machine learning-based systems

To explore the automated classification of MGnify studies as CC-related or not, we implemented two supervised ML methods: logistic regression and XGBoost. These algorithms were evaluated across three distinct training datasets designed to capture different textual features from the MGnify studies. The first, referred to as the *combined super-vector* dataset, is a term frequency matrix based on NER results. It includes all entities from the CC-dictionary that appeared in at least one study. The second, the *condensed* dataset, is a more compact version containing only entities that are both informative, i.e., helpful for distinguishing CC-related from non-CC studies and frequently occurring across multiple studies. The third dataset, called *embeddings*, represents each MGnify training study as a 768-dimensional dense vector produced by the RoBERTa-base language model^49^, which is pretrained on large-scale biomedical and scientific text. All three datasets were used to train both the logistic regression and the XGBoost ML models. Performance evaluation was conducted using repeated k-fold cross-validation (Supplementary Table S2) to ensure robust results. The training part of the corpus was used to calculate performance metrics. Final model selection was based on F1 scores (Supplementary Table S2), allowing identification of the best-performing ML method-dataset combination for accurately classifying CC-related studies.

### Instruction-tuned LLM system

To assess the capabilities of LLMs in classifying MGnify studies as CC-related or not, we evaluated nine instruction-tuned LLMs deployed via the Ollama platform on the LifeWatch ERIC AI-server infrastructure, which consists of a 4 × NVIDIA A100 GPU node. The tested models included: Llama 3.1 8B, Llama 3.3 70B, Mistral NeMo 12B, Mistral Small 22B, Phi-4 14B, Qwen2.5 14B, Qwen2.5 32B, Qwen2.5 72B, and Qwen3 30B A3B (all in quantized q8_0 and instruct versions). For the complete model specifications, see Supplementary Table S3. Prompt engineering was employed to guide the models effectively, including the use of Chain-of-Thought (CoT) prompting techniques, which instruct models to generate reasoned and explainable outputs.

Each prompt was designed to elicit a justification for the classification decision. It also required a structured final answer in JSON format, specifying custom fields such as “explanation” for the brief justification and “answer” for the classification result (yes or no), enabling automated response parsing and evaluation. A simplified version of the LLM final prompts is presented below. Τhe full prompt version, including the JSON-related snippets, is available in the Supplementary Material: D. LLM full prompts.

Prompt4: *Is this text related to climate change directly? Instructions: If in the text is mentioned that climate change (CC) influences a microbiome (MB) community or microbial process (CC—*> *MB) or if microbial communities/processes contribute to Climate Change (MB—*> CC). Both are considered CC-related in this context, meaning that you will mark this text as climate-change-related if either or both is stated. You can type a short explanation for your answer and then answer strictly with ‘***yes***’ (if related) or ‘***no***’ (if unrelated) in the next line. If you are unsure, answer ‘***no***’.

Prompt5: You are a climate change expert. I will provide you with a text describing a microbiome study, and your job is to assess if the study is related to climate change. A study is defined as related to climate change if it analyzes how climate change affects a microbiome or microbiome process, or if it analyzes how a microbiome or microbiome process affects climate change. For each study, you should first provide your explanation and reasoning, followed by your final answer, which must be ***yes** or **no**.

To reduce the randomness in outputs from LLMs, which are autoregressive (generating text one token at a time based on the preceding context), and particularly from lower-parameter models, we performed five independent runs per study for each model. A study was considered CC-related only if at least three out of five responses concluded with a “yes”. Stricter scenarios requiring four, or even five, out of five “yes” responses were also explored.

### Evaluation and comparison of systems

To enable a direct and fair comparison between the ML and instruction-tuned LLM classification systems, we constructed a validation set from the CCMRI corpus. The corpus was divided into 60% training and 40% evaluation, yielding 1,057 aquatic studies for training and 704 for evaluation, as well as 351 terrestrial studies for training and 234 for evaluation. For studies with “related” entries in MGnify, each study and all recursively related studies were assigned to the same partition. This was implemented using a breadth-first search (BFS) traversal to propagate partition assignments through the network of related studies, eliminating potential bias and ensuring a more consistent and reliable dataset.

From the training portion, 117 terrestrial and 352 aquatic studies were randomly selected to form a common validation set, leaving 234 terrestrial and 705 aquatic studies for training the ML models. This validation set was used to obtain preliminary performance estimates for both LLMs and ML models before conducting the final evaluation on the held-out evaluation set.

Among the ML approaches tested, logistic regression trained on the combined super-vector dataset yielded the highest overall performance (Supplementary Table S2) and was selected for comparison with the LLMs, in order to identify the optimal classification method (see Results & discussion).

For the final evaluation of both ML and LLM approaches, the predictions of these classifiers were compared against the reference labels assigned during the manual curation of the CCMRI evaluation corpus. This evaluation was performed separately for terrestrial and aquatic studies (Supplementary Figures S1, S2), as well as on the combined dataset (Fig. 3). Key performance metrics, including accuracy, precision, recall, specificity, and F1 score, were computed to enable consistent and unbiased comparison across all systems. The definitions for these metrics can be found in Supplementary Material: B. Evaluation metric definition.

In addition to these metrics, threshold analysis was conducted on the logistic regression model to explore the trade-offs between precision and recall at various decision thresholds. This analysis enabled the generation of Receiver Operating Characteristic (ROC) and Precision-Recall (PR) curves^50–53^, which are presented alongside the corresponding LLM metrics **(**Fig. 3). Integrating both types of systems into a single set of visualizations provided a comprehensive overview of their relative classification performance under consistent evaluation conditions.

### LLM-based classification of MGnify studies

To screen the remaining 2830 MGnify studies (i.e., except the CCMRI corpus ones), we applied the LLM-based classification pipeline (Fig. 2, purple-filled-arrow path) using the Qwen3 30B A3B model with prompt4, a combination that was selected as the optimal balance between classification performance and computational efficiency (Fig. 3, Performance comparison of study classification methods). The 2830 studies were formatted into a TSV file using a local copy of MGnify data (see Data retrieval), with each line representing a single study containing the available textual data for classification. The text from each study was incorporated into our prompt4 (see previous section and Supplementary Material: D. LLM full prompts). The algorithm presented the LLM with the text for each MGnify study independently. The model returned output in JSON format containing three fields: Study ID, Prompt, and Response. The Response field contained two subfields: explanation (model justification) and answer “yes” or “no” for CC relevance. Each study was evaluated across five independent runs, and the majority voting scheme of at least three yes responses out of five was used to define the CC-related studies. The 81 out of 2830 studies that were classified as CC-related underwent the manual inspection described next.

### Manual inspection and publishing of CC related studies

Once a microbiome study dataset has been processed and potentially CC-related studies have been shortlisted, the shortlisted studies undergo manual inspection; those confirmed as CC-related are then published and made publicly available. The shortlisting (classification) step is performed on a back-end server, while manual curation and publishing take place on the front-end. A back-end daemon automatically communicates candidate CC-related studies via email to the inspection platform, which is implemented as a tailored WordPress blog. Possible CC-related studies are received and converted into draft blog posts, enabling CCMRI curators (acting as WordPress moderators) to review, justify, and decide whether a study is actually CC-related. Confirmed studies are then published through the CCMRI portal, which renders them accessible to the wider community. Both CCMRI curators and portal visitors are provided with additional functionalities through the WordPress platform. These are described in detail in the Results & discussion section.

From a technical perspective, the system operates on a virtual machine server equipped with a 4-core processor and 8 GB of memory, hosting WordPress version 6.8.2 (configured with a maximum of 512 MB RAM, PHP version 8.2.0, and MySQL version 10.4.31-MariaDB). Mailing list management is supported by SYMPA v6.2.40, hosted as a separate service by the Hellenic Centre for Marine Research.

### Periodic update module

The CCMRI platform implements a daemon process (once every two weeks) to automate the retrieval, integration, and classification of MGnify study data. This process begins with the systematic download of all textual information associated with each study.

After that, the daemon cross-checks each study against a continuously updated registry of previously curated and classified studies. If a study has been updated since its last processing, then it is automatically re-downloaded to ensure analyses use the most recent data. Following, the LLM classifies the newly acquired study set and identifies the CC-related ones. These studies (including the pertinent LLMs justifications) are then forwarded by a daemon via email to the WordPress platform, where they are integrated into the web portal (Fig. 2). Manual inspection and publishing of the confirmed CC-related studies can follow (see Database curation and platform update in the Results & discussion section).

## Supplementary Information

Below is the link to the electronic supplementary material.

Supplementary Material 1

## Data Availability

The CCMRI corpus used in this study is publicly available at https://github.com/lab42open-team/ccmri_corpus. This repository contains the corpus created for the CCMRI project. It includes data from MGnify and PubMed (subject to their restrictions), as well as the curation guidelines. The CCMRI database content can be retrieved dynamically via the *Download CC-related studies (in TSV format)* button, available in the CCMRI portal “Download” tab (https://ccmri.hcmr.gr/download). The same functionality is also accessible via the following call to the CCMRI Application Programming Interface (API): https://ccmri.hcmr.gr/?export_posts_tsv_api=1. To maximise its utility, the CCMRI API also supports filtering by study identifier and study category (e.g., https://ccmri.hcmr.gr/?export_posts_tsv_api=1&study_id=MGYS00004048, https://ccmri.hcmr.gr/?export_posts_tsv_api=1&category=methanotrophy, respectively).

## Abbreviations

BFO: Basic Formal Ontology
BFS: Breadth-first search
CC: Climate change
CCMRI: Climate change metagenomic record index
CoT: Chain-of-thought
CSO: Climate System Ontology
ENVO: Environment Ontology
ERIC: European Research Infrastructure Consortium
GO: Gene Ontology
IAA: Inter annotator agreement
LLM: Large language model
LR: Logistic regression
MB: Microbiome
ML: Machine learning
NER: Named entity recognition
PR: Precision-recall
ROC: Receiver operating characteristic
TSV: Tab separated values

## Code availability

The source code used for data gathering, dictionary construction, and study classification (including ML and LLM-based models) is available at the https://github.com/lab42open-team/ccmri_public repository (under the BSD 2-Clause “Simplified” License).
