# Supplementary material for: CCMRI: a classification and curated database of climate change-related microbiome studies
Source: Sci Rep. 2026 May 6;16:20829. doi: 10.1038/s41598-026-51914-z (PMC13338393; doi:10.1038/s41598-026-51914-z)
Supplement: Supplementary file 1 — Supplementary Material 1 [file 41598_2026_51914_MOESM1_ESM.docx]

# Suppleme[n](https://www.zotero.org/google-docs/?broken=AkCyc5)tary Material

## A. CCMRI corpus Inter-annotator agreement (IAA)

| Annotator 1 | Annotator 2 | Cohen’s kappa(κ) | Po | Pe |
| --- | --- | --- | --- | --- |
| Konstantinos | Evangelos | 0.822013 | 0.9789 | 0.881452 |

**Table S1.** Inter-annotator agreement (IAA) for the CCMRI corpus. The table reports the observed agreement (Po), the expected agreement by chance (Pe), and Cohen’s kappa coefficient (κ), calculated as κ = (Po - Pe) / (1 - Pe). Agreement was assessed between two independent curators (Konstantinos and Evangelos). A κ value above 0.80 indicates almost perfect agreement, confirming the reliability and consistency of the manual curation process.

## B. Evaluation metric definition

To assess classification performance, we computed metrics including accuracy, precision, recall, specificity, sensitivity, and F1 score [a]. These metrics quantify the overall correctness of predictions, the ability to correctly identify relevant studies, and the balance between false positives and false negatives for both ML and LLM classification methods.


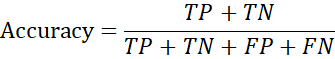


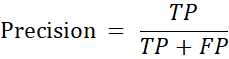


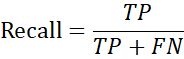


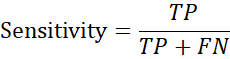


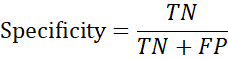


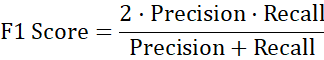


**Reference**

[a] Olson, David L.; and Delen, Dursun (2008); Advanced Data Mining Techniques, Springer, 1st edition (February 1, 2008), page 138, ISBN 3-540-76916-1 (note: in this page, sensitivity and specificity are mentioned as true positive rate, and true negative rate, respectively)

## C. PR and ROC curves for ML and LLMs classification systems - Aquatic and terrestrial datasets


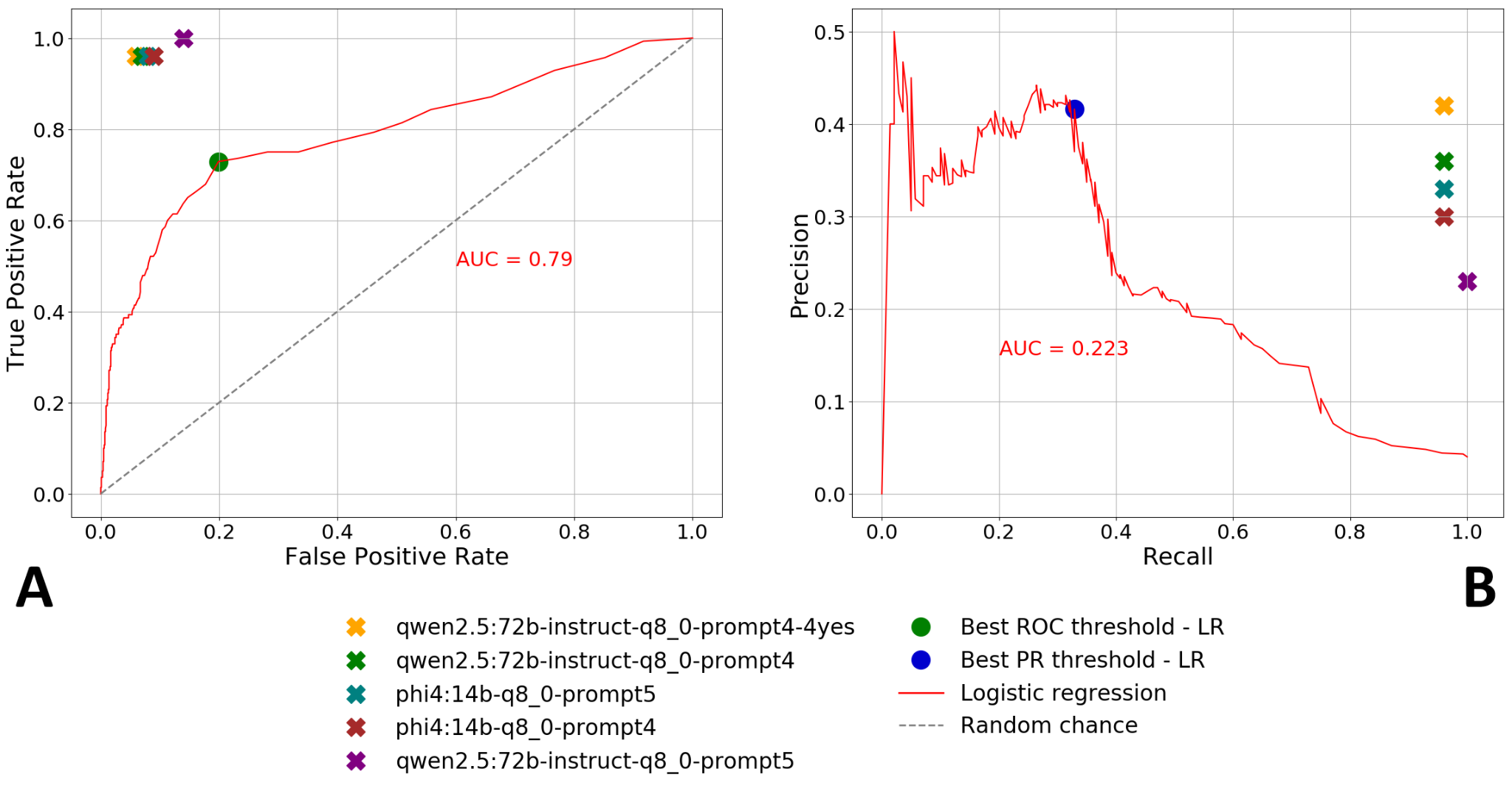


**Figure S1.** Receiver Operating Characteristic (ROC; left) and Precision-Recall (PR; right) curves for the aquatic held-out dataset, comparing the performance of all classification systems. The logistic regression model (red curve) was evaluated through threshold analysis, computing scores across multiple decision thresholds (up to n-1, where n is the number of MGnify studies in the dataset). The optimal ROC and PR thresholds for logistic regression (LR) are marked with green and fluorescent green circles, respectively. The LLM models are represented as individual points, each corresponding to their performance metrics (aggregated over three runs per study using majority voting). ‘4yes’ = requiring four out of five ‘yes’ votes in majority voting. These results provide a comparative overview of the classification capabilities of both traditional ML and instruction-tuned LLM classifiers.s


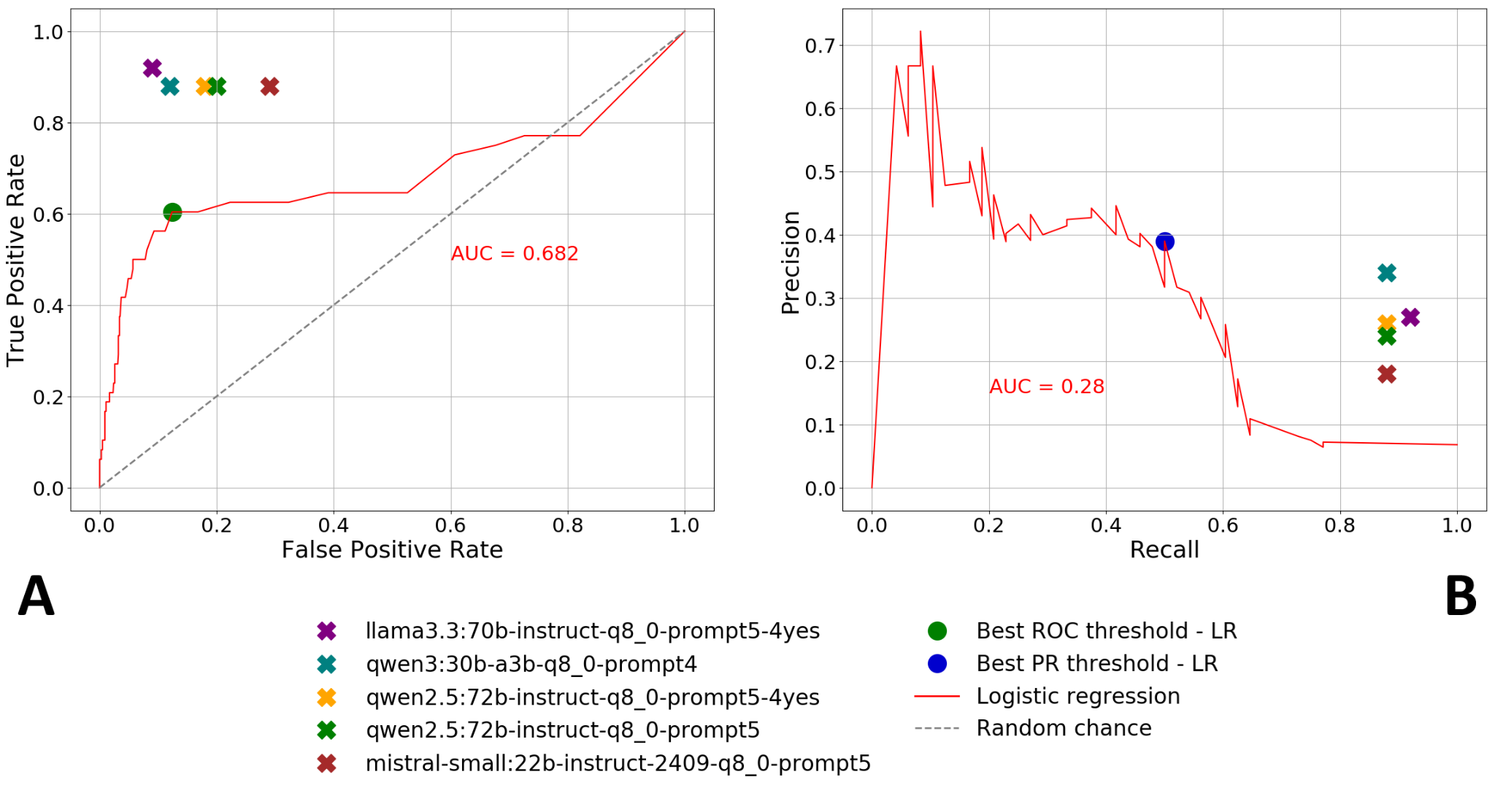


**Figure S2.** Receiver Operating Characteristic (ROC; A) and Precision-Recall (PR; B) curves for the terrestrial held-out dataset, comparing the performance of all classification systems. The logistic regression model (red curve) was evaluated through threshold analysis, computing scores across multiple decision thresholds (up to *n*-*1*, where *n* is the number of MGnify studies in the dataset). The optimal ROC and PR thresholds for logistic regression (LR) are marked with green and fluorescent green circles, respectively. The LLM models are represented as individual points, each corresponding to their performance metrics (aggregated over three runs per study using majority voting). These results provide a comparative overview of the classification capabilities of both traditional ML and instruction-tuned LLM classifiers.

## D. LLM full prompts

**prompt4**

Is this text related to climate change directly? Instructions: If in the text is mentioned that climate change (CC) influences a microbiome (MB) community or microbial process (CC -> MB) or if microbial communities/processes contribute to Climate Change (MB -> CC). Both are considered CC-related in this context, meaning that you will mark this text as climate-change-related if either or both is stated. You can type a short explanation for your answer and then answer strictly with '***yes***' (if related) or '***no***' (if unrelated) in the next line. If you are unsure, answer '***no***'.

Text: study text

Please provide your response in JSON format with the following structure:

{

"explanation": "<short explanation>",

"answer": "<***yes*** or ***no***>"

}

Only return a valid JSON object.

**prompt5**

You are a climate change expert. I will provide you with a text describing a microbiome study, and your job is to assess if the study is related to climate change. A study is defined as related to climate change if it analyzes how climate change affects a microbiome or microbiome process, or if it analyzes how a microbiome or microbiome process affects climate change. For each study, you should first provide your explanation and reasoning, followed by your final answer, which must be ***yes** or **no**.

Text: study text

Please provide your response in JSON format with the following structure:

{

"explanation": "<short explanation>",

"answer": "<***yes*** or ***no***>"

}

Only return a valid JSON object.

## E. Curation platform

**
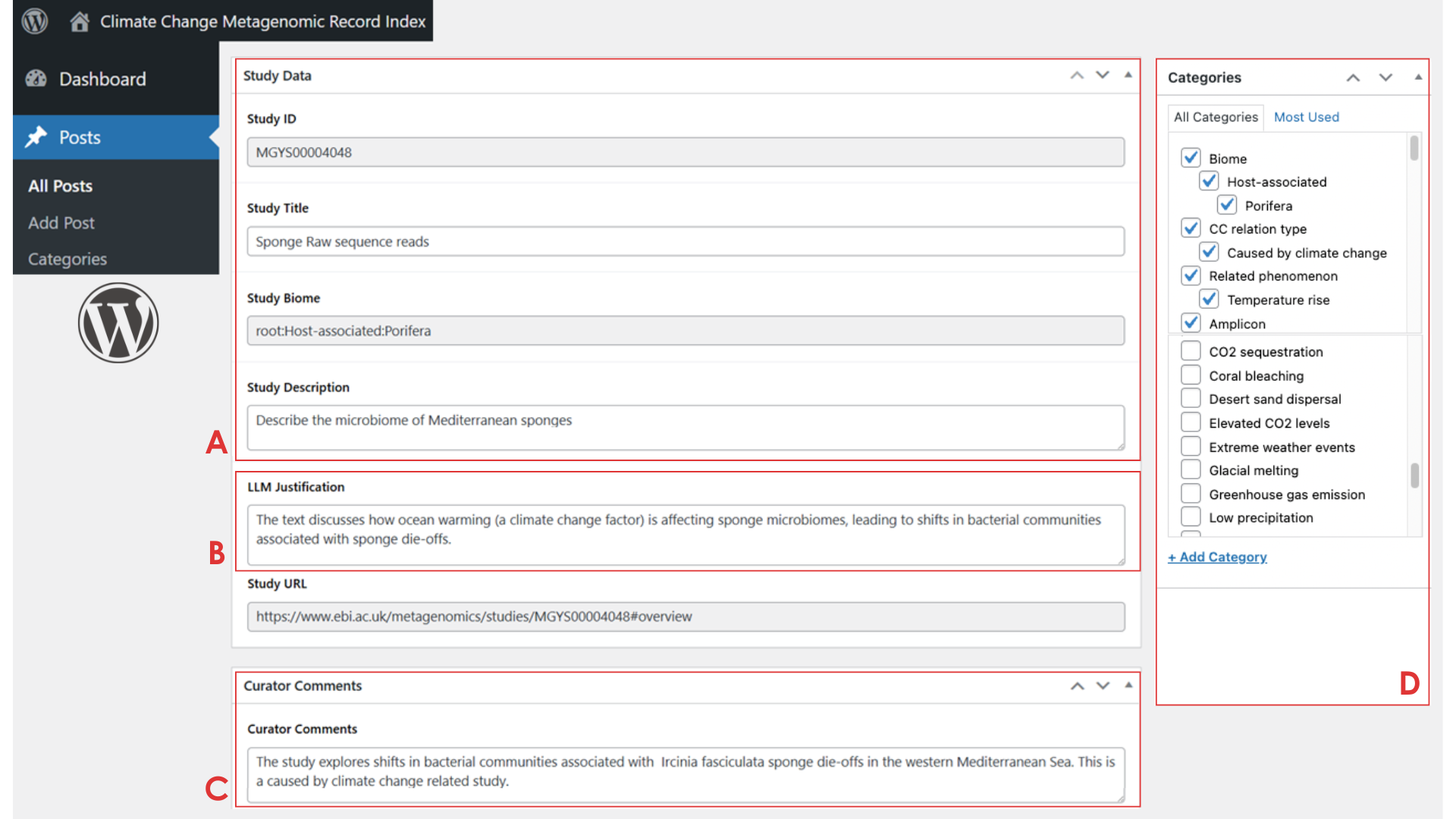
**

**Figure S3.** The CCMRI curation platform: each MGnify study is rendered as a separate WordPress post displaying available metadata (A), the LLM-generated justification for why it was flagged as CC-related (B), and a comment field for curator observations (C). Curators use standardized guidelines and structured checkboxes (D) to verify studies and annotate CC relations and associated environmental phenomena (right). For illustration purposes some of the components shown are arranged differently from how they appear on the web page.


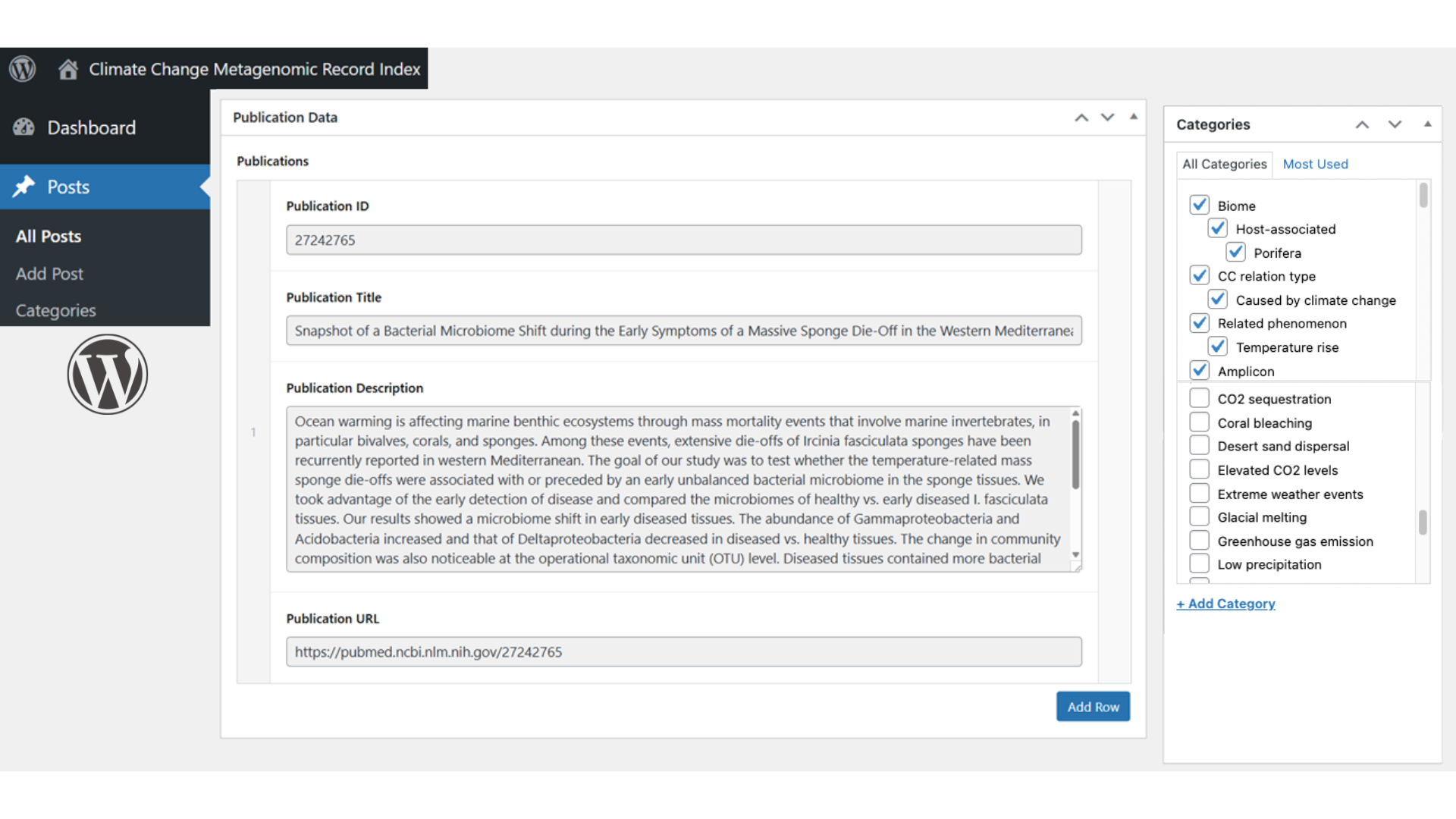


**Figure S4.** Each MGnify study in the curation platform also includes information from related PubMed publications, which include PMID, title, abstract and URL. Once everything is checked by the curator and the study is CC-related, the post can be published on the CCMRI web portal. For illustration purposes, some of the components shown are arranged differently from how they appear on the web page.

## F. Performance metrics of ML methods and training datasets used

| **Classification Method** | **Training dataset** | **K-Folds** | **Repetitions** | **Accuracy** | **Precision** | **Recall** | **Specificity** | **F1 Score** |
| --- | --- | --- | --- | --- | --- | --- | --- | --- |
| Logistic regression | Combined super vector | 3 | 1000 | 0.93 | 0.73 | 0.35 | 0.99 | 0.47 |
| XGBoost | Combined super vector | 3 | 1000 | 0.92 | 0.6 | 0.24 | 0.98 | 0.35 |
| Logistic regression | Condensed vector | 3 | 1000 | 0.92 | 0.55 | 0.12 | 0.99 | 0.2 |
| XGBoost | Condensed vector | 3 | 1000 | 0.92 | 0.63 | 0.24 | 0.99 | 0.35 |
| Logistic regression | Embeddings vector | 3 | 1000 | 0.91 | 0.0 | 0.0 | 1.0 | - |
| XGBoost | Embeddings vector | 3 | 1000 | 0.92 | 0.42 | 0.07 | 1.0 | 0.13 |

**Table S2.** Performance of logistic regression and XGBoost across different datasets (Combined super vector, Condensed vector, and Embeddings). Each model was evaluated using k-fold cross-validation repeated 1000 times (k=3)

## G. LLM Specifications

| **Large Language Model** | **Ollama version ID** | **Ollama URL** | **Installation  Date** |
| --- | --- | --- | --- |
| llama3.1:8b-instruct-q8_0 | b158ded76fa0 | <https://ollama.com/library/llama3.1:8b-instruct-q8_0> | 14-Jan-25 |
| llama3.3:70b-instruct-q8_0 | d5b5e1b84868 | <https://ollama.com/library/llama3.3:70b-instruct-q8_0> | 15-Jan-25 |
| mistral-nemo:12b-instruct-2407-q8_0 | b91eec34730f | <https://ollama.com/library/mistral-nemo:12b-instruct-2407-q8_0> | 29-Jan-25 |
| mistral-small:22b-instruct-2409-q8_0 | ebe30125ec3c | <https://ollama.com/library/mistral-small:22b-instruct-2409-q8_0> | 29-Jan-25 |
| phi4:14b-q8_0 | 310d366232f4 | <https://ollama.com/library/phi4:14b-q8_0> | 10-Feb-25 |
| qwen2.5:14b-instruct-q8_0 | 985c5f25dfe9 | <https://ollama.com/library/qwen2.5:14b-instruct-q8_0> | 15-Jan-25 |
| qwen2.5:32b-instruct-q8_0 | 378290543760 | <https://ollama.com/library/qwen2.5:32b-instruct-q8_0> | 29-Jan-25 |
| qwen2.5:72b-instruct-q8_0 | 23f2cb48bb9a | <https://ollama.com/library/qwen2.5:72b-instruct-q8_0> | 15-Jan-25 |
| qwen3:30b-a3b-q8_0 | 15ec148cb960 | <https://ollama.com/library/qwen3:30b-a3b-q8_0> | 12-May-25 |

**Table S3**. The specification of the LLM models used in this project, including their complete name, their Ollama version identifier and web page, as well as their installation date.

## H. CCMRI workflow figure icons source attribution

- “web site”, <https://thenounproject.com/icon/web-site-6812794/>, by Anwar Hossain, BD
- “database”, <https://thenounproject.com/icon/database-5079143/>, by Alzam
- “text file”, <https://thenounproject.com/icon/text-file-7417501/>, by Creative Art
- “terminal”, <https://thenounproject.com/icon/terminal-4782764/>, by Saepul Nahwan, ID
- “reading a book”, <https://thenounproject.com/icon/reading-a-book-7739938/>, by Agan24, ID
- Aquatic and terrestrial biome icons: MGnify (<https://www.ebi.ac.uk/metagenomics>)
